# Integrating HIV prevention services into care settings for people with opioid use disorder (OUD): a study protocol for implementation strategy development and modeling

**DOI:** 10.1186/s43058-025-00782-1

**Published:** 2025-09-01

**Authors:** Mofan Gu, Ruben G. Martinez, Hannah Parent, Brandon D. L. Marshall, Justin Berk, A. Rani Elwy, Philip A. Chan, Jun Tao

**Affiliations:** 1 Division of Infectious Diseases, The Miriam Hospital, Providence, RI USA; 2 Department of Psychiatry and Human Behavior, The Warren Alpert Medical School of Brown University, Providence, RI USA; 3 Department of Epidemiology, Brown University School of Public Health, Providence, RI USA; 4 Department of Medicine, The Warren Alpert Medical School of Brown University, Providence, RI USA; 5 Department of Pediatrics, Rhode Island Hospital, Providence, RI USA; 6 Department of Behavioral and Social Sciences, Brown University School of Public Health, Providence, RI USA

**Keywords:** HIV prevention, OUD (opioid use disorder), Care integration, APCD (All-Payer Claims Data), ABM (agent-based model), Implementation science, (EBQI) Evidence-based quality improvement

## Abstract

**Background:**

The overlapping epidemics of opioid use disorder (OUD) and HIV present a critical public health challenge. Although people with OUD frequently engage with healthcare settings, uptake of HIV prevention services such as pre-exposure prophylaxis (PrEP) remains low. Integrating HIV prevention into routine OUD care could reduce new infections, but scalable, evidence-based strategies are lacking. Rhode Island offers a unique opportunity to design and evaluate such strategies using its robust data infrastructure and high OUD burden.

**Methods:**

We will conduct a three-phase, sequential implementation study. In Aim 1, we will use the Rhode Island All-Payer Claims Database and State Emergency Department Database data to identify healthcare engagement patterns and gaps in HIV prevention service delivery among people with OUD, including rates of HIV screening, PrEP use, and medications for OUD uptake, across settings from 2012 to 2022. In Aim 2, we will convene a series of five stakeholder-engaged evidence-based quality improvement panels—including with providers, policymakers, and people with lived experience—to co-develop implementation strategies tailored to each care setting (i.e., primary care, mental health clinics, emergency department, and opioid use treatment centers). Finally, in Aim 3, we will develop an agent-based model (ABM) to simulate the population-level effect of implementation strategies developed for each care setting (as identified in Aim 2). The ABM will project outcomes such as HIV incidence, cases averted, and number needed to treat (NNT) over 5- and 10-year horizons under various scenarios. Model parameters will be based on literature and findings from Aim 1. Outputs from the ABM will be used to prioritize feasible, high-impact strategies for future real-world implementation.

**Discussion:**

This study addresses critical gaps in HIV prevention for people with OUD by combining claims-based analysis, evidence-based quality improvement, and agent-based modeling. By leveraging real-world data and engaging diverse stakeholders, the study aims to generate actionable strategies tailored to clinical settings. Findings will inform future implementation efforts in Rhode Island and other jurisdictions facing overlapping HIV and opioid epidemics.

**Trial registration:**

This study does not meet the World Health Organization’s definition of a clinical trial and, therefore, was not registered.

**Supplementary Information:**

The online version contains supplementary material available at 10.1186/s43058-025-00782-1.

Contributions to the literature

- This study is the first to leverage statewide All-Payer Claims and Emergency Department data to identify real-world gaps in HIV prevention and medication for OUD uptake—capturing patterns across insured and uninsured populations, and offering critical insights into opportunities for feasible, targeted implementation strategies.
- Collaborative, evidence-based quality improvement ensures that strategies are co-designed and tailored to the operational realities of clinical settings, enhancing their potential for real-world adoption and sustainability.
- Agent-based modeling enables simultaneous testing of multiple intervention strategies, reducing the time and resources required for sequential trials, while identifying actionable guidance for real-world implementation and scale-up.

## Background

An estimated 2.7 million people have opioid use disorder (OUD) in the United States (US), and more than 105,000 overdose deaths occurred in 2023 [1]. As the opioid overdose epidemic has worsened, there has been growing concern about opioid use fueling the transmission of infectious diseases, including HIV and hepatitis C virus. An estimated 2.6% of the US population has a history of injection drug use, further underscoring the importance of studying links between OUD and HIV [2]. Opioid use has been associated with several HIV outbreaks in the US since 2015, and approximately 2300 new HIV infections were due to injection drug use in 2022 [3–6]. Despite the availability of effective HIV prevention and intervention strategies, many people with OUD do not receive these strategies in the places where they receive care, highlighting a pressing implementation gap [7].

### Barriers to HIV prevention in OUD care

There are several highly effective, evidence-based public health approaches for HIV prevention: HIV screening, treatment as prevention (TasP), pre-exposure prophylaxis (PrEP), and medications for opioid use disorder (MOUD), including buprenorphine, methadone, and naltrexone. Despite the higher risk of HIV among people with OUD, routine incorporation of HIV prevention services into OUD patient care is remarkably low. Merely 0.8% of emergency department (ED) visits in 2020 and 1.09% of primary care visits from 2009 to 2016 included an HIV test [6, 8]. Additionally, less than a third of patients with OUD are screened for HIV during ED visits, missing a key opportunity for early HIV detection and management [9]. The integration of OUD screening and provisions of MOUD in primary care and EDs has similarly not met expectations due to inadequate provider training, provider reluctance, and limited infrastructure and logistical constraints [10–12]. The implementation of PrEP programs in real-world settings like primary care, EDs, and substance use treatment centers has also been limited. Although one-third of patients in EDs qualified for PrEP under the current Centers for Disease Control and Prevention (CDC) guidelines, only 1.2%-2.2% initiated PrEP as a result of their ED visits [13]. Only 6% of primary care providers have ever prescribed PrEP [14]. Overall, the implementation of HIV prevention services into healthcare settings is complicated by a spectrum of social determinants of health (SDH), such as socio-economic status, stigma, and cultural beliefs at the social level, and other issues including insufficient healthcare infrastructure, insurance challenges, and legal barriers at the structural level [7]. At the provider level, discomfort, stigma, and reluctance to prescribe HIV prevention medications also remain key barriers [12, 15, 16]. Despite the availability of educational resources and simplified prescribing protocols, some providers still avoid offering PrEP and/or MOUD due to persistent biases, limited confidence in managing complex social needs, or concern about ‘enabling’ risky behavior [12, 15, 16].

People with OUD frequently encounter the healthcare system through EDs, primary care clinics, substance use treatment centers, and mental health clinics [17, 18]. Approximately one in every 80 ED visits may be due to opioid-related health issues [19]. The 3-year prevalence of OUD among primary care patients was 0.7–1.4% across seven US health systems between 2014 and 2019 [20, 21]. Approximately one-fifth of people with OUD may also seek out treatment at substance use treatment centers [22, 23]. A significant portion, nearly 60%, of those with OUD also suffer from mild to severe mental illnesses [24]. People with OUD could benefit from an integrated healthcare approach due to their multifaceted health needs, including OUD treatment, mental health care, and HIV prevention; however, the reality is that the healthcare system is fragmented—services like MOUD, mental health services, and HIV prevention care are often delivered across different settings. This disconnection leads to missed opportunities for early intervention and makes it challenging for patients with OUD and/or mental illnesses to remain engaged in PrEP care if they are not concurrently receiving treatment [25, 26].

Rhode Island presents a unique opportunity to address these barriers in HIV prevention among people with OUD through the innovative use of population-level administrative data. The state’s All-Payer Claims Database (APCD), coupled with the State Emergency Department Database (SEDD), enables a comprehensive assessment of insured and uninsured individuals across diverse clinical settings, which could provide important insights into real-world healthcare engagement and service utilization, and offers a foundation for designing and implementing scalable, data-driven interventions.

### Implementation science as a promising approach

Incorporating HIV prevention approaches into a comprehensive public health strategy not only addresses the immediate health needs of at-risk populations, but also contributes significantly to the broader goal of HIV elimination. Currently, there is no comprehensive assessment of the frequency and pattern of healthcare seeking behaviors among people with OUD, which could help inform tailored implementation strategies that involve stakeholder engagement, capacity building, and the creation of context-specific HIV prevention services and MOUD delivery models, determining the optimal locations, appropriate providers (nurses or physicians or others), and the most effective methods for HIV prevention services and MOUD [27, 28].

Implementation science is the study and application of methods to integrate evidence-based practices and policies into routine healthcare [29]. Implementation studies often identify and address multi-level determinants (barriers and facilitators), develop implementation strategies, and evaluate the effectiveness of these strategies in addressing implementation outcomes [29]. Developing implementation strategies that are feasible, specific, and effective is a cornerstone of overcoming multi-level barriers and ensuring successful adoption and sustainment of interventions in HIV and OUD care [28, 30]. One promising approach to developing implementation strategies is through an Evidence-Based Quality Improvement (EBQI) model. EBQI fosters multidisciplinary collaboration, lived experience, and integration of evidence-based change strategies to adapt evidence-based practices to local contexts [31, 32]. The EBQI model has demonstrated effectiveness in adapting clinical practices and fostering strong partnerships between researchers and clinicians, making it a promising framework for developing feasible, specific, and effective implementation strategies to overcome barriers to integrating HIV prevention services in healthcare settings where people with OUD seek care [33, 34].

### Agent-based modeling

Evaluating the effectiveness of implementation strategies in real-world settings can be time-consuming and resource intensive [35]. Agent-based modeling is a powerful tool to test complex approaches, such as implementation strategies, by simulating a collection of decision-making individuals (called “agents”) within a system. These autonomous agents interact with each other and their environment, allowing researchers to explore how individuals of a system behave as a function of individual characteristics and interactions with each other and the environment [36]. Agent-based models (ABMs) have several advantages for studying implementation strategies, including: 1) allowing bi-directional and non-linear interactions between individuals, organizations, and environmental factors; 2) defining dynamic decision-making processes; 3) simulating processes like adoption, negative/positive feedback loops, and relational structures such as social networks; 4) estimating population-level outcomes while accounting for individual-level heterogeneity; and 5) acting as "intervention testing laboratories" when real-world experimentation is infeasible or too costly [37–41]. Several studies in areas such as tobacco control and HIV prevention have successfully employed ABM to estimate the effect of complex interventions to guide implementation decisions for improving the delivery of evidence-based practices [42–46]. However, most of these models focused on transmission dynamics (i.e., how diseases spread within a population) rather than evaluation of the implementation strategies for integrating HIV prevention services into OUD care. ABM offers an innovative approach to test such strategies, especially when informed by stakeholder input and real-world data.

### The current study

This study leverages several innovative approaches to develop and test implementation strategies that overcome barriers to integrating HIV prevention into care settings where people with OUD are already seeking services. Aim 1 analyzes healthcare and HIV prevention service utilization patterns among people with OUD using statewide claims and emergency department (ED) data to identify multi-level barriers in HIV prevention and MOUD uptake among people with OUD. Aim 2 develops feasible and context-specific implementation strategies through stakeholder engagement using implementation science methods nested within an EBQI model. Aim 3 evaluates the population-level impact of developed implementation strategies through agent-based modeling.

## Methods

We followed the *StaRI* (Standards for Reporting Implementation Studies) guidelines when preparing this protocol to ensure completeness and transparency [47]. The completed checklist is included as an Additional file.

### Aim 1 – Claims-based utilization analysis

#### Study design

Aim 1 will employ a retrospective cohort study design to quantify healthcare engagement and HIV prevention service use among people with OUD in RI. This analysis will use Rhode Island All-Payer Claims Database (APCD) and ED data to identify service utilization patterns across different clinical settings from 2012 to 2022. These findings will inform subsequent strategy development and simulation testing in OUD care in Aims 2 and 3.

#### Data sources

We will use two primary data sources to capture insured and uninsured individuals in RI:

- Rhode Island (RI) All-Payer Claims Database (APCD) includes longitudinal medical and pharmacy claims, participant demographics and enrollment information, and provider characteristics data from private insurers, Medicare, and Medicaid (Table 1). APCD data are updated monthly and reflect over 90% of the insured population in RI (based on our preliminary analysis). We will use APCD data between 2012 and 2022 to evaluate trends over time.
- Rhode Island State Emergency Department Database (SEDD): Captures encounter-level data for ED visits, including those involving uninsured individuals (estimated 5.0% of RI population, based on 2022 data) [48]. While SEDD lacks unique patient identifiers and pharmacy/provider details, it complements APCD by providing a more complete picture of ED utilization among patients who may be excluded from claims-based analyses. Because SEDD does not allow for longitudinal tracking of individuals across visits, these data will be used to characterize encounter-level trends among the uninsured population. APCD and SEDD will not be linked at the individual level; however, each will be analyzed separately but interpreted together to capture utilization across the full population of people with OUD.

**Table 1:** Data variables for statistical analysis in Aim 1

Demographic data	Medical care information	Provider information
Age Sex ZIP Code City Insurance information Relationship to health policyholder Internal unique identifier	Date of service Type of service Location of service Date prescription(s) filled ICD-9/10 diagnostic codes National Drug Code Prescription refill indicator Pharmacy ID Inpatient and outpatient claims Mental health claims	Practice Attending provider name Prescribing provider name Type Primary care indicator Specialty Address ZIP Code City Pharmacy ID

#### Study population

The study population will consist of people who are: 18 years of age or older, residing in Rhode Island during health insurance enrollment, and presented evidence of OUD (see OUD criteria below), between 2012 and 2022. To ensure robustness of findings, we will include only individuals who had 6 months of continuous health insurance coverage after their first OUD-related claim within a calendar year. People with OUD will be identified in APCD using one or more of the following:

- International Classification of Diseases (ICD-9/10) diagnosis codes for OUD, encompassing diagnostic codes for opioid use, opioid abuse, and opioid dependence.
- Healthcare Common Procedure Coding System (HCPCS) and Current Procedural Terminology (CPT) codes for OUD-related services, including OUD treatment administration (e.g., injection and oral medication administration).
- National Drug Codes (NDCs) for MOUD, including various forms (e.g., injection and medication), dosages, strengths, and formulations (e.g., branded and generic) of methadone, buprenorphine, and naltrexone.

The study cohort will include individuals with at least one qualifying encounter or prescription during the study period. A supplemental table (Table S1) will provide the complete list of codes used for cohort identification.

#### Outcome measures

**Fig. 1:**
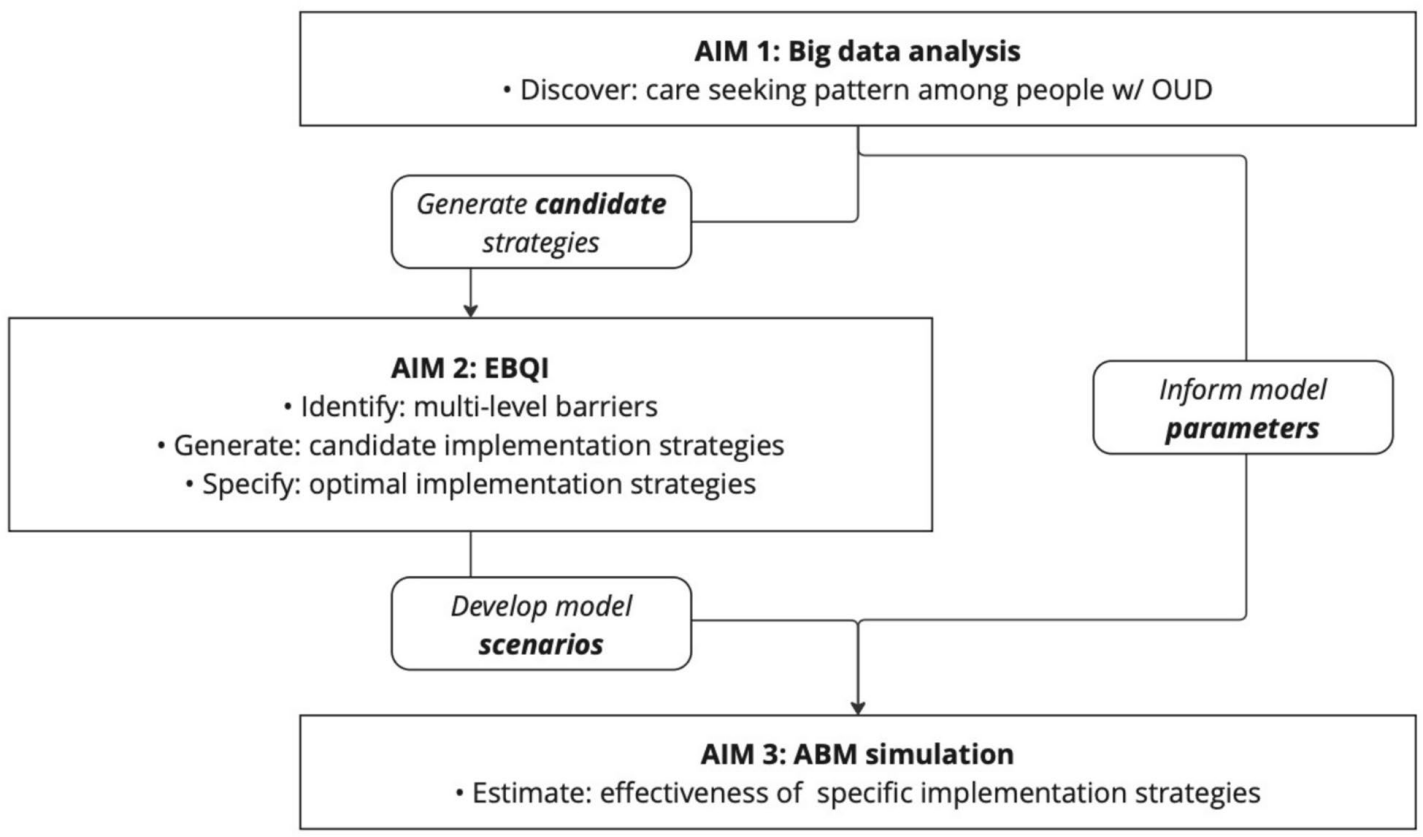
Overview of three aims in our study

We will assess healthcare utilization across four types of care settings, including ED, primary care, substance use treatment center, and mental health service facility (Fig. 1).

- ED visits will be identified in APCD using HCPCS (99,281–99,285) and revenue codes (0450–0459, 0981). Uninsured ED visits will be identified in SEDD using OUD and MOUD diagnosis codes. Given the lack of unique identifiers in SEDD, each record represents a single encounter.
- Primary care visits will be identified using CPT codes for outpatient visits (9201–99215, 99,381–99,397), HCPCS code T1015, and provider specialty codes (01, 08, 11, 19, 31, 38, 50, 84, 97) indicating primary care (e.g., internal medicine, family medicine, nurse practitioners, physician assistants).
- Substance use treatment visits will be identified via CPT codes for substance use services (e.g., H0015, H0018, H0020), including MOUD-related services. Claims will be restricted to facilities verified as SAMHSA-regulated Opioid Treatment Programs (OTPs) in Rhode Island. We recognize that office-based MOUD services are increasingly provided in primary care and other outpatient settings; however, these visits will be captured under the primary care setting definition.
- Mental health service visits will be identified using CPT codes (90,791–90853, 99,404) and provider taxonomy for psychiatry, psychology, and behavioral health providers.

In particular, we will assess receipt of HIV prevention services, including HIV testing and PrEP, as well as MOUD. We will use CPT codes (86,689, 86,701–86703, 87,534–87,536, 87,390) and HCPCS codes (G0432–G0435) to identify claims for HIV testing. PrEP use will be defined as having a prescription for a 30-day or longer supply of tenofovir/emtricitabine or tenofovir alafenamide/emtricitabine, without a concurrent diagnosis of HIV or hepatitis B virus. MOUD receipt will use the definitions stated above, based on NDC and procedure codes. All utilization outcomes will be summarized and stratified by care setting and insurance type where applicable. We will also explore temporal trends across the study period.

#### Covariates

Where feasible, we will link individual claims to neighborhood-level characteristics using residential ZIP codes or census tracts. Data from the American Community Survey (ACS) and other Census Bureau resources will be used to assign area-level indicators of socioeconomic status, including median household income, educational attainment, and housing instability. We will also examine area-level incarceration rates and opioid-related mortality where available. These variables will be used descriptively and incorporated into regression models as contextual covariates. Examples of data variables to be used in our analysis are summarized in Table 1.

#### Analysis plan

**Fig. 2:**
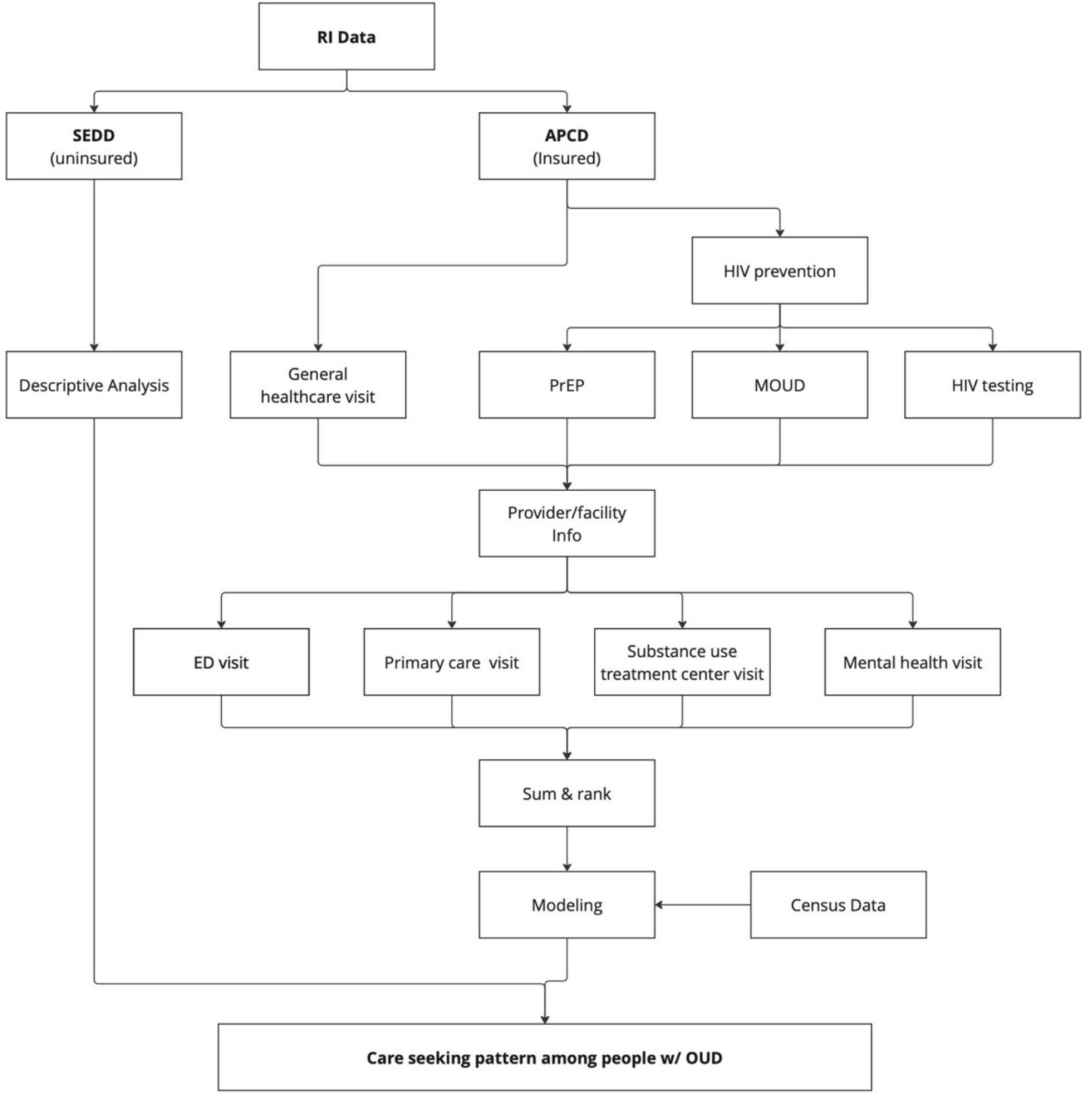
Flowchart of retrospective data analysis in Aim 1

Statistical analyses include descriptive analysis and regression modeling (Fig. 2).

In descriptive analysis, we will summarize annual frequencies and rates of healthcare utilization and HIV prevention service uptake by setting and insurance status. Results will be stratified by age group, gender, race/ethnicity, and geographic region. Statistical tests (e.g., Chi-square, t-test, and ANOVA) and trend analyses will be used to examine differences in service utilization across years and subgroups.

In regression modeling, we will fit multivariable logistic regression models to identify individual- and area-level factors that are associated with receipt of HIV testing, PrEP uptake and retention, and MOUD initiation and retention (defined as continuous MOUD coverage for 180 days, permitting gaps no longer than 7 days) [49]. Covariates will include demographic characteristics (age, gender, race/ethnicity), insurance type, healthcare setting, and SDH indicators (e.g., housing instability, incarceration). Area-level variables from the U.S. Census Bureau (i.e., American Community Survey) will be linked to each individual via zip codes. If data structure allows, we will implement multilevel models to account for clustering at the geographic level. We will report adjusted odds ratios (aORs) with 95% confidence intervals. We will conduct several sensitivity analyses with alternate definitions of PrEP use and retention (e.g., 60-days of supply, and allowing for various lengths of gaps between refills). All analyses will be conducted using SAS 9.4. Statistical significance will be assessed at the *p* < 0.05 level unless otherwise specified.

### Aim 2 – Strategy development via EBQI panels

#### Study design

The objective of Aim 2 is to collaboratively develop and refine implementation strategies for integrating HIV prevention services and MOUD into real-world healthcare settings serving people with OUD in RI. This aim builds upon the findings of Aim 1 and existing literature, and leverages a EBQI model, engaging a multidisciplinary workgroup of key stakeholders through a series of structured panels.

#### Stakeholder recruitment

We will recruit a diverse group of 30 stakeholders across three key groups: healthcare providers, policy leaders, and people with lived experience (PWLE) of OUD. Specifically, we plan to include 10 healthcare providers (e.g., physicians and nurses) from various specialties such as emergency medicine, primary care, mental health, addiction medicine, and HIV treatment. Another 10 members will be public health and policy-level decision-makers involved in OUD service delivery or health system planning, including leadership from state agencies, social services, and healthcare organizations. Finally, we will recruit 10 community advocates with lived experience (current or past) of OUD, and/or involvement in harm reduction services. Participants will be recruited through existing networks such as the Rhode Island Overdose Prevention & Intervention Task Force, nonprofit organizations, and the Brown University Health network. All stakeholders will be invited to participate in a series of five EBQI panel sessions. PWLE will contribute perspectives on barriers to accessing HIV prevention services, stigma, and preferences for service delivery. Provider and policymaker participants will focus on identifying regulatory, clinical, and logistical barriers and facilitators to implementation. All participants will provide informed consent, and ethical oversight will be maintained throughout. Each participant will be compensated $200 per session.

#### EBQI procedures

The five in-person EBQI sessions are designed to follow a logical sequence that begins with sharing findings from Aim 1 and culminates in a finalized strategy package to be tested in Aim 3. EBQI activities will be guided by existing implementation frameworks and methods. The Consolidated Framework for Implementation Research (CFIR) will inform structured discussions around multi-level barriers in the implementation of HIV prevention care [50, 51]. Prior to the first session, the research team will prepare a summary of Aim 1 findings and an updated literature review of existing implementation strategies that have been used for care integration. These materials will be reviewed and refined in collaboration with stakeholders during the EBQI process. Each EBQI session, facilitated by the research team, will last approximately two hours.

##### Session 1: Introduction and presentation of aim 1 findings

This session will establish a shared understanding of the project's goals and introduce participants to the findings from Aim 1 and literature review. The research team will start the session with an introduction of the workgroup (stakeholders and research team members), followed by a presentation of healthcare utilization patterns and service gaps. Stakeholders will provide feedback through a member-checking process, ensuring findings reflect real-world experience. The session will conclude with an initial discussion about promising care settings for integrating HIV prevention services, and their potential impacts.

##### Session 2: Identification of implementation strategies

Prior to Session 2, the research team will use the barriers identified through Aim 1 analysis and synthesis of Session 1 to identify a “menu” of promising implementation strategies using the CFIR-ERIC matching tool [52]. The primary purpose of Session 2 is to bring this “menu” of implementation strategies to stakeholders to identify the most impactful strategies and to generate novel strategies. Participants will be grouped by role to ensure equitable input from PWLE, healthcare providers, and policymakers. Each group will map strategies to care settings and present recommendations to the full panel for refinement.

##### Session 3: Refinement and operationalization

In Session 3, participants will further refine selected strategies (from Session 2) by discussing feasibility, scalability, and necessary resources. They will define key implementation components, including action, roles, delivery methods, required resources, and target outcomes [53]. The workgroup will develop timeline and processes for integration into current workflows, as well as key performance indicators (KPIs) to guide future implementation evaluation. Select KPIs—such as service uptake rates, frequency of care engagement, and provider adherence—will also inform the parameterization of the agent-based model (Aim 3), ensuring that simulation scenarios are grounded in stakeholder-defined expectations. Special emphasis will be placed on addressing the needs of uninsured populations and accounting for social determinants of health.

##### Session 4: Review of draft strategies

The research team will first present the refined strategies developed from Session 3. Stakeholders will review the strategies for alignment with project goals, practical relevance, and cultural competence, and identify outstanding implementation barriers and feasibility concerns. Feedback will be incorporated in real time to make final adjustments.

##### Session 5: Finalization of strategy package

In the final session, the research team will present finalized implementation strategies. The workgroup will rank these strategies on their importance and feasibility, and confirm consensus on the selected implementation strategies to be evaluated through agent-based modeling in Aim 3. If consensus cannot be reached, structured decision-making techniques (e.g., breakout discussions, ranked-choice voting) will be used. And lastly, the workgroup will discuss potential steps beyond the study, including considerations of real-world deployment, sustainability, and post-implementation evaluation. While actual implementation is not part of the current study, such discussion could inform potential operationalization in future research and inform local implementation efforts.

#### Analysis plan

The sessions will be audio recorded, transcribed using NVivo, and analyzed using rapid qualitative analysis [54]. Rapid qualitative analysis will allow the research team to balance rigor of analysis with the need to rapidly synthesize data within sessions to inform each subsequent session. The process will include validity checks through independent coding by multiple members. Key insights will inform the refinement of implementation strategies, with all processes adhering to ethical guidelines to maintain confidentiality for participant contributions.

#### Output: implementation strategy package

**Fig. 3:**
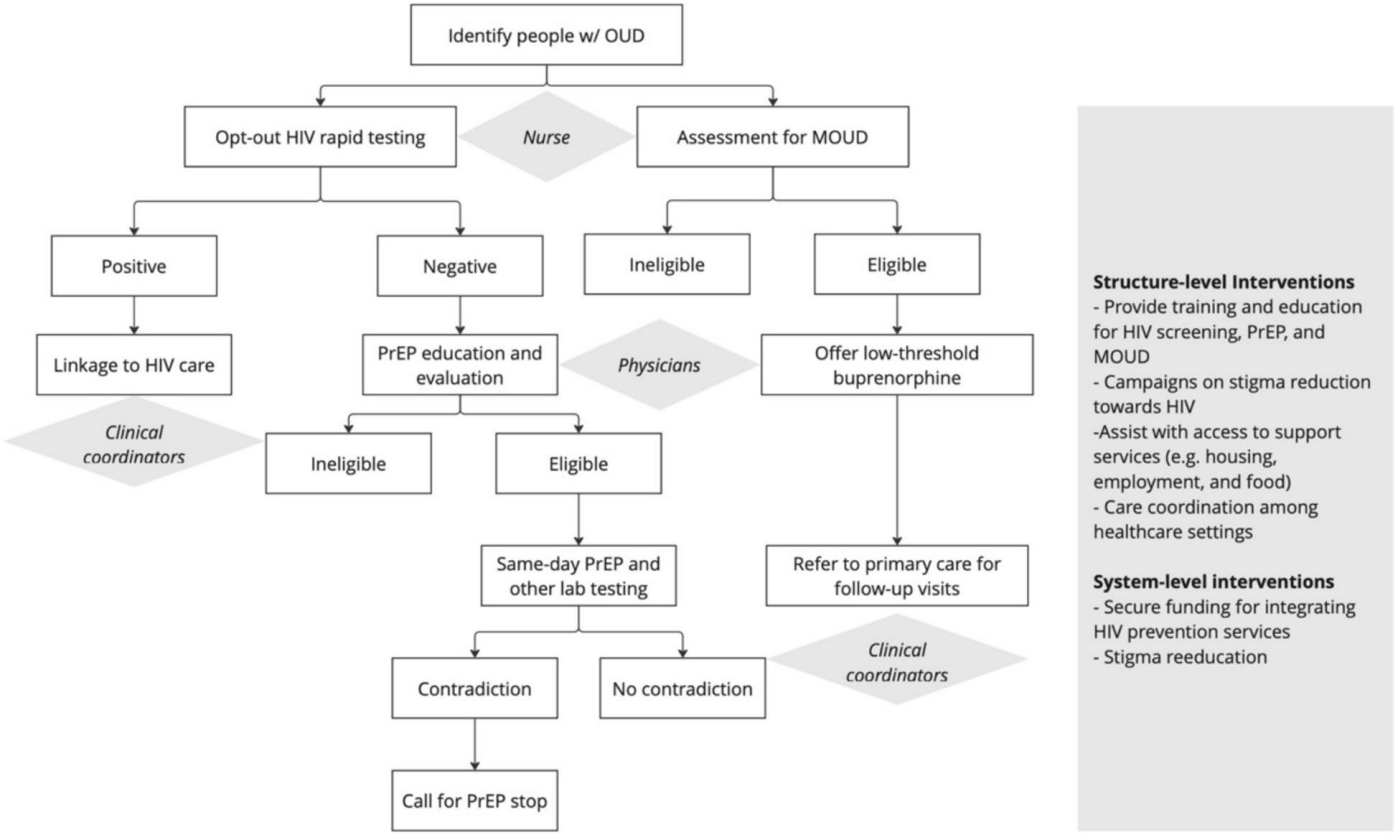
A hypothetical example of implementation strategy in an ED setting

Implementation strategies are defined as methods or techniques used to facilitate the adoption, implementation, and sustainability of a clinical program or practice, including provider training and decision support, intervention-specific tool kits, checklists, formal practice protocols and guidelines, and economic, fiscal, and regulatory strategies [51, 53]. For each selected implementation strategy, we will define and operationalize each implementation strategy needed to address multilevel barriers by describing the actors, action, targets of the action, temporality, doses, and implementation outcomes [53]. An example of implementation strategy is shown in Fig. 3. We anticipate finalizing up to three care-setting-specific strategy scenarios for simulation testing in Aim 3.

### Aim 3 – Simulation modeling via agent-based modeling

#### Study design

The objective of Aim 3 is to develop and apply an agent-based model (ABM) to simulate HIV transmission, healthcare engagement, and the effect(s) of hypothetical implementation strategies on key health outcomes among people with OUD in Rhode Island. The model will project outcomes over 5- and 10-year horizons, assessing the potential reach and effectiveness of tailored implementation approaches, identified in Aim 2, prior to real-world piloting.

#### Model framework

The ABM will simulate a “virtual society” of people in Rhode Island with OUD, representing dynamic, heterogeneous agents interacting through injecting and/or sexual networks. The model will build upon the TITAN model (prior open-source simulation developed by Dr. Marshall’s team), and incorporate healthcare-seeking behaviors across various care settings (ED, primary care clinic, substance use treatment center, and mental health clinic), and interactions between provider agents and OUD patient agents during HIV prevention care [55]. The final model will be implemented in Python™, and managed by a trained research programmer with ABM expertise. Our model will operate in discrete (monthly) time steps, with stochastic transitions between states and interactions governed by empirically derived probabilities. The model will simulate the delivery of HIV prevention and MOUD services within care settings and assess how implementation strategies modify these probabilities to influence health outcomes (e.g., HIV incidence trajectories) in Rhode Island.

#### Model input

Model parameters will be drawn from three sources: administrative data from Aim 1 (e.g., healthcare usage patterns by demographic subgroup), stakeholder-derived strategy components from Aim 2 (e.g., settings and delivery mechanisms for HIV prevention services), and published literature on HIV natural history and behavioral dynamics. A repository of parameter values, assumptions, and data sources will be created to guide calibration and facilitate reproducibility.

Agents will have attributes that evolve over time, and allow for bidirectional interactions between agents and their environment. At each discrete time step, agents will be connected to each other in a population-based contact network. Network edges (representing relationships) will be formed, retained, or broken over the lifetime of the simulation, representing partnership acquisition and dissolution in a dynamic contact network. This network of interactions will represent the primary pathway(s) for HIV transmission within the agent population. Examples of model parameters are listed in Table 2.

**Table 2:** Example parameters in agent-based models to assess effectiveness of intervention package(s)

**Patient-level**
Acceptance of rapid HIV testing, PrEP, and MOUD Possibility of HIV-negative results Chance of being interested in PrEP and MOUD Possibility of PrEP and MOUD eligible Chance of receiving a PrEP/MOUD prescription Possibility of picking up PrEP/MOUD Estimated time on PrEP/MOUD Possibility of seeking care in EDs, primary care, mental health, and substance use centers Patterns of key SDH
**Provider-level**
Chance of receiving PrEP/MOUD education and training Possibility of receiving a certificate for a PrEP/MOUD prescription Chance of conducting HIV and PrEP counseling and MOUD assessment Possibility of conducting a PrEP/MOUD evaluation Chance of prescribing PrEP/MOUD Possibility of referring for follow-up visits
**System-level**
Chance of funding to support HIV prevention services and MOUD Effectiveness of care coordination between care settings

At model initialization, “agents” of people with OUD will be randomly assigned endogenous traits based on information collected during Aim 1 and the literature.^37^ These discrete state variables include demographics (age, sex, race, ethnicity), risk behavior profiles (injection and non-injection drug use, sexual risk behavior), clinical characteristics (HIV status, PrEP use, MOUD status, healthcare usage), and key SDHs (housing, incarceration, insurance status, employment, sex work involvement). Agents representing people with OUD will be subject to a probability of death due to opioid overdose, HIV-related diseases, or other causes that will be impacted by their gender, drug use status, incarceration status, and the usage of healthcare in general. Meanwhile, agents representing healthcare providers will also be randomly assigned endogenous characteristics including: demographics, role (e.g., nurse, physician, clinical coordinator), attitudes towards HIV and OUD, knowledge (of HIV, PrEP, MOUD), and previous history of PrEP prescription. Agents representing healthcare providers will be subject to the probability of stopping their medical practice in the selected care setting due to retirement, resignation, and death.

The simulation will also include a set of environmental parameters/variables (e.g., how clinics, labs, and pharmacies are connected geographically or operationally) and the availability of resources (e.g., staffing capacity, pharmacy access, and laboratory infrastructure). These environmental factors will influence access to care, delays in treatment, and referral patterns, and are critical to accurately modeling real-world constraints in health service delivery.

#### Outcome definition

The base model will represent the status quo—current levels of HIV prevention service delivery across different care settings, as established through Aim 1. Using Python™, we will evaluate the effect of each proposed implementation strategy (developed in Aim 2) on multiple outcome measures over 5- and 10-year projections.

Key outcomes will include projected HIV incidence across the population, as well as the number of new HIV cases averted or caused relative to the base model. We will also assess changes in healthcare utilization patterns and quantify uptake of PrEP and MOUD by setting. For each intervention scenario, we will calculate the number needed to treat (NNT)—defined as the number of HIV prevention service encounters or MOUD prescriptions required to prevent a single HIV infection over a specified time horizon. And lastly, the model will assess the population-level impact of integrating HIV prevention services into OUD care. These outcomes will guide the selection of the most effective and feasible strategy package for real-world implementation and inform the prioritization of approaches for future pilot testing and scale-up.

#### Model calibration and validation

Model calibration will follow a multi-step approach. First, we will create a repository of context-specific parameters, documenting data sources, values, and their temporal variability. This parameter repository will facilitate identification of input uncertainty and guide systematic model development. Next, we will evaluate the robustness of simulation outcomes under varying assumptions through a structured stress test of three rule sets: (1) a core rule set, based on strong empirical evidence; (2) an expanded rule set, incorporating additional but less well-supported behavioral and structural processes; and (3) a randomized rule set, intended to assess the effects of confounding or unanticipated dynamics.

After defining the rule sets, we will calibrate the model using Approximate Bayesian Computation (ABC) to reproduce observed historical trends, including HIV incidence and outcomes derived in Aim 1 such as ED utilization among people with OUD. Model outputs will be validated by comparing simulated and empirical HIV incidence trends in Rhode Island using Euclidean distance metrics. Calibration will be iteratively refined until model outputs closely match observed data. Because the model includes numerous stochastic components, all simulations will employ Markov Chain Monte Carlo methods to produce robust distributions of outcome estimates. For each rule set, the model will be executed 1,000 times to estimate central tendencies and variances. All simulations will be run on a high-performance computing cluster to ensure computational efficiency and scalability.

#### Sensitivity analysis

To manage the inherent uncertainty in model parameters, we will conduct a range of sensitivity analyses. We will identify cases where small input changes—particularly those related to the improved intervention targeting—produce large variations in the outcome (HIV cases averted). Such cases suggest the presence of a critical variable that merits increased attention. Each parameter in the model will be varied systematically across a range in a series of one-way sensitivity analyses. Those that exhibit the largest magnitude of influence will be varied together in multi-way sensitivity analyses. We plan to visualize findings with a “tornado diagram.”

## Discussion

### Importance of a multiphase approach

This protocol outlines a multiphase, multi-method implementation science study to optimize the delivery of HIV prevention services among people with OUD in Rhode Island. By integrating retrospective analysis of big data (Aim 1), stakeholder-driven strategy development (Aim 2), and agent-based modeling simulation (Aim 3), this study aims to generate actionable, context-specific implementation strategies to effectively integrate HIV prevention services—including HIV screening, PrEP, and MOUD—into healthcare settings.

### Anticipated contributions

Our approach addresses critical gaps in the integration of HIV prevention services within systems that serve people with OUD. While the public health urgency of the HIV/OUD syndemic is well-established, real-world implementation of evidence-based interventions among people with OUD remains limited. This study offers several key contributions: (1) a comprehensive analysis of insured (APCD) and uninsured (SEDD) data to identify missed opportunities for HIV prevention; (2) use of an EBQI process to co-design strategies with healthcare providers, policymakers, and individuals with lived experience; and (3) application of ABM to test and predict impact of implementation scenarios. Each aim builds upon one another, producing a validated, context-specific final strategy that is ready for real-world implementation.

### Implementation barriers

Implementation of HIV prevention and MOUD services is influenced by multilevel barriers, including individual-level SDH (e.g., mental health disorders, housing instability, food insecurity, lack of insurance, and financial challenges), and structural level factors (e.g., limited provider capacity, institutional fragmentation, stigma, low PrEP prescribing rate, policy restrictions on PrEP coverage). Moreover, while current PrEP delivery models mainly target men who have sex with men (MSM), they often do not address the needs of people with OUD, who also face barriers in accessing MOUD [27, 56]. To successfully integrate HIV prevention services in diverse care settings, implementation science is needed to overcome these multi-level barriers and design feasible implementation strategies to increase the uptake of both HIV prevention and evidence-based OUD treatments. By engaging stakeholders across the care system from people with OUD experience to policymakers, our EBQI process is designed to develop context-specific solutions that address these barriers. Furthermore, the ABM will allow us to test the feasibility and expected effectiveness of these strategies in silico before piloting them in real-world care settings.

### Strengths: Sequential design, real-world data, multi-method approach

This study offers multiple strengths that enhance its scientific and translational value. First, it leverages comprehensive, statewide claims and emergency department data to generate a robust understanding of healthcare engagement among a high-priority, often underrepresented population. Second, the integration of stakeholder-driven input through EBQI ensures that proposed implementation strategies are contextually relevant and grounded in real-world practice. Third, the use of agent-based modeling adds a powerful simulation layer to forecast the long-term impact of competing strategies. Together, these components provide a rigorous, multi-method framework with strong potential to guide scalable and sustainable implementation in diverse care settings.

### Limitations: Claims data, modeling assumptions, generalizability

There are several limitations of this proposed study. First, claims data may not fully capture behavioral risk factors, patient preferences, or quality of care. Second, the SEDD dataset does not include unique identifiers, limiting longitudinal tracking of uninsured individuals, and we may not capture uninsured OUD patients outside of ED settings, limiting the generalizability of our study. Third, the generalizability of our study findings are further limited by the varied policies on OUD and HIV prevention across different states. However, the methodology could inform similar studies in other states.

### Next Steps

This study represents a pioneering, multidisciplinary big-data approach, integrating mathematical modeling and implementation science to address complex health issues and evaluate implementation strategies. We plan to develop a future R01 project for actual implementation study based on this study, focusing on HIV prevention and MOUD in key settings to maximize impact. Our research, utilizing the APCDs and SEDD data, will have broad relevance, particularly for states heavily impacted by OUD and HIV issues. Importantly, these data-driven insights can guide public health agencies to prioritize limited resources by identifying which healthcare settings, strategies, and populations offer the greatest potential impact for integrated HIV prevention and MOUD delivery.

## Supplementary Information

Supplementary Material 1.

Supplementary Material 2.

## Data Availability

The datasets analyzed during the current study, including APCD and SEDD, are not publicly available due to strict data use agreements and access restrictions. Individuals interested in using these data must submit a formal application through the appropriate data custodians. However, the implementation strategies protocol developed in Specific Aim 2, along with the full agent-based models (ABM) and simulation outputs, will be made publicly available to support transparency and future research.

## Abbreviations

ABM: Agent-based model
ABC: Approximate Bayesian computation
APCD: All-Payer Claims Database
CFIR: Consolidated Framework for Implementation Research
CPT: Current Procedural Terminology
EBQI: Evidence-Based Quality Improvement
ED: Emergency department
ERIC: Expert Recommendations for Implementing Change
HCPCS: Healthcare Common Procedure Coding System
HIV: Human immunodeficiency virus
ICD: International Classification of Diseases
MCMC: Markov chain Monte Carlo
MOUD: Medications for opioid use disorder
NDC: National Drug Code
NNT: Number needed to treat
OUD: Opioid use disorder
PrEP: Pre-exposure prophylaxis
RI: Rhode Island
SDH: Social determinants of health
SEDD: State Emergency Department Database

## References

[1] National Institute on Drug Abuse. Drug Overdose Deaths: Facts and Figures 2024. Available from: https://nida.nih.gov/research-topics/trends-statistics/overdose-death-rates#Fig1.

[2] Lansky A, Finlayson T, Johnson C, Holtzman D, Wejnert C, Mitsch A, et al. Estimating the Number of Persons Who Inject Drugs in the United States by Meta-Analysis to Calculate National Rates of HIV and Hepatitis C Virus Infections. PLoS ONE. 2014;9(5): e97596. 24840662 10.1371/journal.pone.0097596 PMC4026524

[3] Centers for Disease Control and Prevention. HIV Surveillance Supplemental Report: Estimated HIV Incidence and Prevalence in the United States, 2018–2022. 2024.

[4] Centers for Disease Control Prevention. Diagnoses of HIV infection in the United States and dependent areas, 2019. HIV surveillance report. 2021;32.

[5] Hershow RB, Wilson S, Bonacci RA, Deutsch-Feldman M, Russell OO, Young S, et al. Notes from the field: HIV outbreak during the COVID-19 Pandemic among persons who inject drugs—Kanawha County, West Virginia, 2019–2021. Morb Mortal Wkly Rep. 2022;71(2):66. 10.15585/mmwr.mm7102a4 PMC8757623 35025854

[6] Peters PJ, Pontones P, Hoover KW, Patel MR, Galang RR, Shields J, et al. HIV Infection Linked to Injection Use of Oxymorphone in Indiana, 2014–2015. N Engl J Med. 2016;375(3):229–39. 27468059 10.1056/NEJMoa1515195

[7] Duffy M, Ghosh A, Geltman A, Mahaniah GK, Higgins-Biddle M, Clark M. Coordinating Systems of Care for HIV and Opioid Use Disorder: A Systematic Review of Enablers and Barriers to Integrated Service Access, and Systems and Tools Required for Implementation. Medical care research and review : MCRR. 2022;79(5):618–39. 34634961 10.1177/10775587211051182 PMC9397399

[8] Clay CE, Hoover KW, Le Guen Y, Bennett CL. Estimates of HIV testing at visits to United States emergency departments. London, England: AIDS. 2024;38(2). 10.1097/QAD.0000000000003750 PMC10842496 37830905

[9] Lyons MS, Chawarski MC, Rothman R, Whiteside L, Cowan E, Richardson LD, et al. Missed Opportunities for HIV and Hepatitis C Screening Among Emergency Department Patients With Untreated Opioid Use Disorder. J Addict Med. 2023;17(2):210–4. 36170184 10.1097/ADM.0000000000001074 PMC10023471

[10] Korthuis PT, McCarty D, Weimer M, Bougatsos C, Blazina I, Zakher B, et al. Primary care–based models for the treatment of opioid use disorder: A scoping review. Ann Intern Med. 2017;166(4):268–78. 27919103 10.7326/M16-2149 PMC5504692

[11] Lagisetty PA, Ross R, Bohnert A, Clay M, Maust DT. Buprenorphine Treatment Divide by Race/Ethnicity and Payment. JAMA Psychiat. 2019;76(9):979–81. 10.1001/jamapsychiatry.2019.0876 PMC6506898 31066881

[12] Cui S, Ding H, Huang X, Wang H, Tang W, Leuba SI, et al. Factors Influencing Clinicians’ Willingness to Prescribe Pre-exposure Prophylaxis for Persons at High Risk of HIV in China: Cross-sectional Online Survey Study. JMIR Public Health Surveill. 2021;7(6): e24235. 34085941 10.2196/24235 PMC8214180

[13] Gormley MA, Nagy TR, Moschella P, Lu Z, Rodriguez J, Roth P. HIV Pre-Exposure Prophylaxis in the Emergency Department: A Systematic Review. Ann Emerg Med. 2023;81(4):468–81. 36117011 10.1016/j.annemergmed.2022.07.015

[14] Raifman J, Nocka K, Galarraga O, Wilson IB, Crowley C, Tao J, et al. Evaluating statewide HIV preexposure prophylaxis implementation using All-Payer Claims Data. Ann Epidemiol. 2020;44:1-7.e2. 32279914 10.1016/j.annepidem.2020.03.003 PMC7252205

[15] Edwards KA, Merlin JS, Webster F, Mackey SC, Darnall BD. Breaking barriers: addressing opioid stigma in chronic pain and opioid use disorder. PAIN. 2025;166(6):1268–73. 10.1097/j.pain.0000000000003475 PMC12066799 39560423

[16] Skaggs P, Bell SB, Scutchfield FD, Robinson LE. Providers’ Stigmas and the Effects on Patients with Opioid Use Disorder: A Scoping Review. J Appalach Health. 2023;4(3):87–102. 38026051 10.13023/jah.0403.06 PMC10655729

[17] Hawk K, McCormack R, Edelman EJ, Coupet E Jr, Toledo N, Gauthier P, et al. Perspectives About Emergency Department Care Encounters Among Adults With Opioid Use Disorder. JAMA Netw Open. 2022;5(1): e2144955. 35076700 10.1001/jamanetworkopen.2021.44955 PMC8790663

[18] Austin EJ, Briggs ES, Ferro L, Barry P, Heald A, Curran GM, Saxon AJ, Fortney J, Ratzliff AD, Williams EC. Integrating Routine Screening for Opioid Use Disorder into Primary Care Settings: Experiences from a National Cohort of Clinics. J Gen Intern Med . 2023;38(2):332–40. 10.1007/s11606-022-07675-2. 10.1007/s11606-022-07675-2 PMC9132563 35614169

[19] Langabeer JR, Stotts AL, Bobrow BJ, Wang HE, Chambers KA, Yatsco AJ, et al. Prevalence and charges of opioid-related visits to U.S. emergency departments. Drug Alcohol Depend. 2021;221:108568. 33578297 10.1016/j.drugalcdep.2021.108568

[20] Boudreau DM, Lapham G, Johnson EA, Bobb JF, Matthews AG, McCormack J, et al. Documented opioid use disorder and its treatment in primary care patients across six US health systems. J Subst Abuse Treat. 2020;112s:41–8. 32220410 10.1016/j.jsat.2020.02.001 PMC7107675

[21] Lapham G, Boudreau DM, Johnson EA, Bobb JF, Matthews AG, McCormack J, et al. Prevalence and treatment of opioid use disorders among primary care patients in six health systems. Drug Alcohol Depend. 2020;207: 107732. 31835068 10.1016/j.drugalcdep.2019.107732 PMC7158756

[22] Saloner B, Karthikeyan S. Changes in Substance Abuse Treatment Use Among Individuals With Opioid Use Disorders in the United States, 2004–2013. JAMA. 2015;314(14):1515–7. 26462001 10.1001/jama.2015.10345

[23] Wu LT, Zhu H, Swartz MS. Treatment utilization among persons with opioid use disorder in the United States. Drug Alcohol Depend. 2016;169:117–27. 27810654 10.1016/j.drugalcdep.2016.10.015 PMC5223737

[24] Novak P, Feder KA, Ali MM, Chen J. Behavioral health treatment utilization among individuals with co-occurring opioid use disorder and mental illness: Evidence from a national survey. J Subst Abuse Treat. 2019;98:47–52. 30665603 10.1016/j.jsat.2018.12.006 PMC6350939

[25] Substance Abuse and Mental Health Services Administration. Prevention and treatment of HIV among people living with substance use and/or mental disorders. 2020.

[26] Substance Abuse and Mental Health Services Administration. Health care systems and substance use disorders. Facing Addiction in America: The Surgeon General's Report on Alcohol, Drugs, and Health: US Department of Health and Human Services, 2016. 28252892

[27] Vanhamel J, Rotsaert A, Reyniers T, Nöstlinger C, Laga M, Van Landeghem E, et al. The current landscape of pre-exposure prophylaxis service delivery models for HIV prevention: a scoping review. BMC Health Serv Res. 2020;20(1):704. 32736626 10.1186/s12913-020-05568-w PMC7395423

[28] Powell BJ, Waltz TJ, Chinman MJ, Damschroder LJ, Smith JL, Matthieu MM, et al. A refined compilation of implementation strategies: results from the Expert Recommendations for Implementing Change (ERIC) project. Implement Sci. 2015;10(1):21. 25889199 10.1186/s13012-015-0209-1 PMC4328074

[29] Bauer MS, Kirchner J. Implementation science: What is it and why should I care? Psychiatry Res. 2020;283: 112376. 31036287 10.1016/j.psychres.2019.04.025

[30] Kirchner JE, Smith JL, Powell BJ, Waltz TJ, Proctor EK. Getting a clinical innovation into practice: An introduction to implementation strategies. Psychiatry Res. 2020;283: 112467. 31488332 10.1016/j.psychres.2019.06.042 PMC7239693

[31] Smith JL, Williams JW Jr, Owen RR, Rubenstein LV, Chaney E. Developing a national dissemination plan for collaborative care for depression: QUERI Series. Implementation science : IS. 2008;3:59. 19117524 10.1186/1748-5908-3-59 PMC2631596

[32] Rubenstein LV, Parker LE, Meredith LS, Altschuler A, dePillis E, Hernandez J, et al. Understanding team-based quality improvement for depression in primary care. Health Serv Res. 2002;37(4):1009–29. 12236381 10.1034/j.1600-0560.2002.63.x PMC1464007

[33] Hempel S, Bolshakova M, Turner BJ, Dinalo J, Rose D, Motala A, et al. Evidence-Based Quality Improvement: a Scoping Review of the Literature. J Gen Intern Med. 2022;37(16):4257–67. 36175760 10.1007/s11606-022-07602-5 PMC9708973

[34] Stockdale SE, Hamilton AB, Bergman AA, Rose DE, Giannitrapani KF, Dresselhaus TR, et al. Assessing fidelity to evidence-based quality improvement as an implementation strategy for patient-centered medical home transformation in the Veterans Health Administration. Implementation science : IS. 2020;15(1):18. 32183873 10.1186/s13012-020-0979-y PMC7079486

[35] Eisman AB, Kilbourne AM, Dopp AR, Saldana L, Eisenberg D. Economic evaluation in implementation science: Making the business case for implementation strategies. Psychiatry Res. 2020;283: 112433. 31202612 10.1016/j.psychres.2019.06.008 PMC6898762

[36] Bonabeau E. Agent-based modeling: Methods and techniques for simulating human systems. Proc Natl Acad Sci. 2002;99(suppl_3):7280–7. 12011407 10.1073/pnas.082080899 PMC128598

[37] Fioretti G. Agent-based simulation models in organization science. Organ Res Methods. 2013;16(2):227–42.

[38] Harrison JR, Lin Z, Carroll GR, Carley KM. Simulation modeling in organizational and management research. Acad Manag Rev. 2007;32(4):1229–45.

[39] Hammond RA. Considerations and best practices in agent-based modeling to inform policy. Assessing the use of agent-based models for tobacco regulation: National Academies Press (US); 2015. 26247084

[40] Hammond RA, Ornstein JT. A model of social influence on body mass index. Ann N Y Acad Sci. 2014;1331(1):34–42. 24528150 10.1111/nyas.12344 PMC4133329

[41] Epstein JM. Why model? J Artif Soc Soc Simul. 2008;11(4):12.

[42] Huang W, Chang CH, Stuart EA, Daumit GL, Wang NY, McGinty EE, et al. Agent-Based Modeling for Implementation Research: An Application to Tobacco Smoking Cessation for Persons with Serious Mental Illness. Implement Res Pract. 2021;2. 10.1177/26334895211010664. 10.1177/26334895211010664 PMC8297792 34308355

[43] Goedel WC, King MRF, Lurie MN, Nunn AS, Chan PA, Marshall BDL. Effect of Racial Inequities in Pre-exposure Prophylaxis Use on Racial Disparities in HIV Incidence Among Men Who Have Sex With Men: A Modeling Study. J Acquir Immune Defic Syndr (1999). 2018;79(3):323–9. 10.1097/QAI.0000000000001817 PMC6342014 30044303

[44] Marshall BD, Paczkowski MM, Seemann L, Tempalski B, Pouget ER, Galea S, et al. A complex systems approach to evaluate HIV prevention in metropolitan areas: preliminary implications for combination intervention strategies. PLoS ONE. 2012;7(9): e44833. 23028637 10.1371/journal.pone.0044833 PMC3441492

[45] Tracy M, Gordis E, Strully K, Marshall BDL, Cerdá M. Applications of agent-based modeling in trauma research. Psychological trauma : theory, research, practice and policy. 2022. 10.1037/tra0001375 PMC10030380 36136775

[46] Zang X, Goedel WC, Bessey SE, Lurie MN, Galea S, Galvani AP, et al. The impact of syringe services program closure on the risk of rebound HIV outbreaks among people who inject drugs: a modeling study. AIDS (London, England). 2022;36(6):881–8. 35212666 10.1097/QAD.0000000000003199 PMC9081164

[47] Pinnock H, Barwick M, Carpenter CR, Eldridge S, Grandes G, Griffiths CJ, et al. Standards for Reporting Implementation Studies (StaRI) Statement. BMJ (Clinical research ed). 2017;356: i6795. 28264797 10.1136/bmj.i6795 PMC5421438

[48] Tolbert J, Drake P, Damico A. Key Facts about the Uninsured Population 2023. Available from: https://www.kff.org/uninsured/issue-brief/key-facts-about-the-uninsured-population/.

[49] Williams AR, Mauro CM, Feng T, Wilson A, Cruz A, Olfson M, et al. Performance Measurement for Opioid Use Disorder Medication Treatment and Care Retention. Am J Psychiatry. 2023;180(6):454–7. 36285405 10.1176/appi.ajp.20220456 PMC10130230

[50] Damschroder LJ, Reardon CM, Lowery JC. The consolidated framework for implementation research (CFIR). Handbook on implementation science: Edward Elgar Publishing; 2020. p. 88–113.

[51] Damschroder LJ, Reardon CM, Widerquist MAO, Lowery J. The updated Consolidated Framework for Implementation Research based on user feedback. Implement Sci. 2022;17(1):75. 36309746 10.1186/s13012-022-01245-0 PMC9617234

[52] CFIR Research Team. CFIR-ERIC Matching Tool. 2025.

[53] Proctor EK, Powell BJ, McMillen JC. Implementation strategies: recommendations for specifying and reporting. Implement Sci. 2013;8(1):1–11. 24289295 10.1186/1748-5908-8-139 PMC3882890

[54] Gale RC, Wu J, Erhardt T, Bounthavong M, Reardon CM, Damschroder LJ, et al. Comparison of rapid vs in-depth qualitative analytic methods from a process evaluation of academic detailing in the Veterans Health Administration. Implement Sci. 2019;14(1):11. 30709368 10.1186/s13012-019-0853-y PMC6359833

[55] Gantenberg JR, King M, Montgomery MC, Galarraga O, Prosperi M, Chan PA, et al. Improving the impact of HIV pre-exposure prophylaxis implementation in small urban centers among men who have sex with men: An agent-based modelling study. PLoS ONE. 2018;13(7): e0199915. 29985949 10.1371/journal.pone.0199915 PMC6037355

[56] Johnson J, Gormley MA, Bentley S, Baldwin C, Bublitz M, Heavner SF, et al. HIV Preexposure Prophylaxis Care Continuum Among Individuals Receiving Medication for Opioid Use Disorder, South Carolina, 2020–2021. Am J Public Health. 2022;112(1):34–7. 34936400 10.2105/AJPH.2021.306566 PMC8713624

