# Supplementary material for: Integrating HIV prevention services into care settings for people with opioid use disorder (OUD): a study protocol for implementation strategy development and modeling
Source: Implement Sci Commun. 2025 Sep 1;6:93. doi: 10.1186/s43058-025-00782-1 (PMC12400686; doi:10.1186/s43058-025-00782-1)
Supplement: Supplementary file 2 — Supplementary Material 2. [file 43058_2025_782_MOESM2_ESM.docx]

**Supplement**

**Supplement Table 1** Codes used to identify claims associated with opioid use disorder (OUD) in administrative claims database, including the All-Payer Claims Database (APCD) and State Emergency Department Database (SEDD) in Rhode Island

| **Opioid use disorder (OUD)** | ICD-9 | 305.50-305.52: Opioid Abuse  304.00-304.02: Opioid-Type Dependence  304.70-304.72: Combinations of Opioid-Type Drug with Any Other Drug Dependence |
| --- | --- | --- |
|  | ICD-10 | F11.10: Opioid abuse, uncomplicated  F11.120-F11.129: Opioid abuse with intoxication  F11.14: Opioid abuse with opioid-induced mood disorder  F11.150-F11.159: Opioid abuse with opioid-induced psychotic disorder  F11.181-F11.188, F11.19: Opioid abuse with opioid-induced disorders  F11.19: Opioid abuse with unspecified opioid-induced disorder  F11.20: Opioid dependence, uncomplicated  F11.220-F11.229: Opioid dependence with intoxication  F11.23: Opioid dependence with withdrawal  F11.24: Opioid dependence with opioid-induced mood disorder  F11.250-F11.259: Opioid dependence with opioid-induced psychotic disorder  F11.281-F11.288: Opioid dependence with other opioid-induced disorders  F11.29: Opioid dependence with unspecified opioid-induced disorder  F11.90: Opioid use, unspecified, uncomplicated  F11.920-922, F11.929: Opioid use, unspecified with intoxication  F11.93: Opioid use, unspecified, with withdrawal.  F11.94: Opioid use, unspecified, with opioid-induced mood disorder.  F11.950-951 Opioid use, unspecified with opioid-induced psychotic disorder  F11.959: Opioid use, unspecified with opioid-induced psychotic disorder, unspecified.  F11.981-F11.988 Opioid use, unspecified with opioid-induced disorders  F11.99: Opioid use, unspecified, with unspecified opioid-induced disorder |
| **Medications for Opioid Use Disorder (MOUD)** | NDC | ***Buprenorphine***  00054017613, 00054017713, 00054018813, 00054018913, 00093537856, 00093537956, 00093572056, 00093572156, 00121101930, 00121203830, 00228315303, 00228315403, 00228315473, 00228315503, 00228315567, 00228315573, 00228315603, 00378092393, 00378092493, 00406053701, 00406053710, 00406053725, 00406053762, 00406054201, 00406054210, 00406054225, 00406054262, 00406192303, 00406192309, 00406192403, 00406192409, 00406800503, 00406802003, 00490005100, 00490005130, 00490005160, 00490005190, 12496120201, 12496120203, 12496120401, 12496120403, 12496120801, 12496120803, 12496121201, 12496121203, 12496127802, 12496128302, 12496130602, 12496131002, 16590066605, 16590066630, 16590066705, 16590066730, 16590066790, 23490927003, 23490927006, 23490927009, 35356000407, 35356000430, 35356055530, 35356055630, 42858050103, 42858050203, 43598090630, 43598090690, 43598090730, 43598090790, 43598090830, 43598090890, 49999039507, 49999039515, 49999039530, 49999063830, 49999063930, 50090157100, 50268014411, 50268014415, 50268014511, 50268014515, 50383028793, 50383029493, 50383092493, 50383093093, 52959030430, 52959074930, 53217013830, 53217024630, 54123011430, 54123090730, 54123091430, 54123092930, 54123095730, 54123098630, 54569549600, 54569573900, 54569573901, 54569573902, 54569639900, 54569640800, 54569657800, 54868570700, 54868570701, 54868570702, 54868570703, 54868570704, 54868575000, 55045378403, 55700014730, 55700018430, 55700030230, 55700030330, 55887031204, 55887031215, 59385001201, 59385001230, 59385001401, 59385001430, 59385001601, 59385001630, 60429058630, 60429058633, 60429058730, 60429058733, 62175045232, 62175045832, 62756045964, 62756045983, 62756046064, 62756046083, 62756096964, 62756096983, 62756097064, 62756097083, 63481016160, 63481020760, 63629403401, 63629403402, 63629403403, 63629409201, 63629409202, 63629507401, 63629712501, 63629712502, 63629712503, 63629712504, 63629712505, 63629712506, 63629712507, 63629712601, 63629712602, 63629712603, 63629712604, 63629712605, 63629712606, 63629712607, 63629712608, 63629727001, 63629727002, 63874108403, 63874108503, 63874117303, 63874117403, 64725093003, 64725093004, 64725192403, 64725192404, 65162041503, 65162041509, 65162041603, 65162041609, 66336001530, 66336001630, 68071138003, 68071151003, 68258299103, 68258299903, 68308020230, 68308020830, 71335035301, 71335035302, 71335035303, 71335035304, 71335035305, 71335035306, 71335035307, 76519117000, 76519117001, 76519117002, 76519117003, 76519117004, 00093537966, 69238120201, 00904700906, 00904701006, 60687062611, 60687063711, 00409215101, 00409215201, 00409215301, 00228315403, 00228315503, 00228315311, 47781035503, 47781035603, 47781035703, 47781035803, 42858060103, 42858060203, 43063066706, 42291017430, 42291017530, 43063018407, 43063018430,  00409201232, 04202317905, 12496010001, 12496010230, 12496010290, 12496010330, 12496010390, 12496010530, 12496010590, 12496010630, 12496010690, 12496030001, 12496030002, 12496030005, 12496075705, 58284010014, 58284020801, 58284021601, 58284022401, 58284023201, 58284026401, 58284029601, 58284022801  ***Naltrexone***  00406117003, 65757030001, 63459030001, 63459030002, 63459030003, 63459030004, 63459030005, 63459030010, 63459030011, 63459030012 |
|  | HCPCS / CPT | ***Methadone***  H0020, G2067, G2076, G2077  ***Buprenorphine***  G2078, J0571, J0572, J0573, J0574, J0575, J0577, J0578, Q9991, Q9992, J0570, G2070, G2071  ***Naltrexone***  J2315, G2080 |
